# COVID-19-related anxiety and lifestyle changes

**DOI:** 10.3389/fpubh.2022.886137

**Published:** 2022-11-01

**Authors:** Song Yi Han, Hye Young Jang, Young Ko

**Affiliations:** ^1^Department of Nursing Science, Sunmoon University, Asan-si, South Korea; ^2^College of Nursing, Hanyang University, Seoul, South Korea; ^3^College of Nursing, Gachon University, Incheon, South Korea

**Keywords:** coronavirus (COVID-19), anxiety, lifestyle, healthy behavior, addictive behavior

## Abstract

This study aimed to identify factors that affect lifestyle changes and focused on coronavirus disease (COVID-19)-related anxiety since the COVID-19 outbreak in South Korea. Data from 213,848 individuals from the 2020 Korean Community Health Survey were analyzed using a complex sampling design. Descriptive statistics, *t*-tests, one-way ANOVA, and multiple regression analyses were performed. Participants reported a high level of COVID-19-related anxiety, with a score of 19.28 out of 25. The score of healthy behavioral change index was −0.51, indicating negative changes in physical activity, dietary habits, and sleep patterns. A slight positive change was reported for addictive behavioral change index, such as smoking and alcohol consumption, at 0.27 scores, indicating a decrease in these behaviors. COVID-19-related anxiety was an important factor that negatively affected health behavior. The high-risk groups that were vulnerable to anxiety included older adults and those who have little social support or few social encounters. Thus, identifying high-risk groups with the potential for worsened health behavior and providing interventions to reduce the anxiety caused by COVID-19 are necessary.

## Introduction

The coronavirus disease (COVID-19) pandemic, which began in 2019, has led to a global public health emergency and has continued to spread. The World Health Organization (WHO) declared a pandemic in March 2020, and as of December 2021, there have been reports of more than 200 million infections and 5 million deaths (1). Recently, as the Omicron variant has spread rapidly, the WHO has emphasized the need for continuous implementation of physical distancing, refraining from non-essential outings, quarantining, and isolating and has requested national cooperation to prevent the spread of COVID-19 (2). Since 2019, many countries including Korea have taken strong measures to prevent and control COVID-19, which has resulted in many changes in daily lives. Changes in health behaviors such as decreased physical activity (3–6), changes in sleeping patterns (3, 6, 7), and increased consumption of an unhealthy diet have been reported (3, 6). Stress and isolation during COVID-19 have also led to an increase in cigarette smoking and alcohol consumption, which both have negative impacts on health (8). These changes in health behaviors will result in various health problems (9). Therefore, identifying high-risk groups with adverse changes in lifestyle behaviors during the COVID-19 pandemic and factors influencing these behaviors is necessary.

The COVID-19 pandemic has been highly associated with psychological problems, such as anxiety and depression. In previous studies, high levels of mental health problems such as depression and anxiety symptoms were reported during COVID-19 (10, 11). Increase in COVID-19-related anxiety with the spread of this novel infectious disease may come from fear of infection, social isolation, physical distancing to prevent transmission, and worsening economic issues (12–14). COVID-19-related anxiety is reportedly higher with advanced age (15–17), low socioeconomic status (18), and low social support (14, 17). High anxiety levels have been attributed to lifestyle changes such as decreased physical activity or quality of sleep (18, 19). In particular, for older adults, high COVID-19-related anxiety is known to decrease health-related quality of life (20).

Previous studies have been conducted to assess changes in health behavior during the COVID-19 pandemic (7) or to examine the relationship between psychological factors, such as depression, stress, and anxiety, and changes in health behavior (4, 8, 18, 19, 21). Some studies have utilized assessment tools for general anxiety instead of those focused on COVID-19 (8, 18, 19), and inconsistent findings have thus been reported on the association between anxiety and lifestyle changes. Moreover, there are limitations in generalizing these findings as the studies involved specific populations (8, 18, 19, 21).

Therefore, using data representative from the Korean population, this study aims to examine the degree of lifestyle changes associated with COVID-19-related anxiety (Aim 1) and to identify factors that affect these lifestyle changes by focusing on COVID-19-related anxiety (Aim 2). We hypothesized that COVID-19-related anxiety would be associated with lifestyle changes. Higher levels of COVID-19-related anxiety would be related with negative changes in health behaviors, which include physical activity, sleep and diet. Addictive behaviors, which include alcohol consumption and smoking, would occurred with higher levels of COVID-19-related anxiety. This study will help to promote positive lifestyle changes and to establish health promotion strategies during the ongoing COVID-19 pandemic.

## Materials and methods

### Study design

This study involved a secondary data analysis of the 2020 Korean Community Health Survey (KCHS) to identify the factors influencing lifestyle changes by focusing on COVID-19-related anxiety since the onset of the COVID-19 pandemic.

### Participants and data

The KCHS has been conducted annually since 2008 at 255 community health centers across the nation among adults aged 19 years or older. This survey was conducted by the Korea Disease Control and Prevention Agency. The questionnaire of this survey was reviewed and finalized by community health survey expert group. Raw data were collected from August 16, 2020, to October 31, 2020. Trained investigators visited household selected as samples and conducted one-on-one computer assisted personal interviews (CAPI). Informed consent was obtained from all subjects before participation. The 2020 KCHS involved 229,269 participants in total. In this study, 213,848 individuals were included in the analysis, excluding subjects with missing values. The data can be obtained in accordance with the regulations on the disclosure procedure of raw data established by the Korea Disease Control and Prevention Agency. This study was conducted with the approval from the institutional review board (IRB) at the author's university (IRB No. 1044396-202111-HR-227-01).

### Measurements

#### Lifestyles

Lifestyle factors were classified into healthy behavior and addictive behavior. We reconstructed these items based on previous studies (7, 8, 18). Each behavioral change was measured as follows.

##### Healthy behavioral change index

The Healthy Behavioral Change Index included physical activity, sleep duration, and unhealthy diet. The following three items were included: “Compared with prior to the COVID-19 pandemic, what kind of changes have you noticed in (1) physical activity, such as walking or exercising (including indoor and outdoor activities), (2) sleep duration, and (3) consumption of instant foods or carbonated beverages?” The response options were as follows: 1 = increased; 2 = similar; 3 = decreased; and 4 = not applicable. The responses were recoded for physical activity and sleep duration: 1 = increased; 0 = similar and not applicable; and −1 = decreased. The responses for unhealthy diet were recoded as follows: 1 = decreased; 0 = similar and not applicable; and −1 = increased. The total score of the above three items was calculated. Total scores ranged from −3 to 3, with higher scores indicating a positive change in healthy behavior.

##### Addictive behavioral change index

The Addictive Behavioral Change Index included smoking and alcohol consumption. The following two items were included: “Compared with prior to the COVID-19 pandemic, what kind of changes have you noticed in your (1) alcohol consumption and (2) smoking?” The response options were as follows: 1 = increased; 2 = similar; 3 = decreased; and 4 = not applicable. These responses were recoded as follows: 1 = decreased; 0 = similar and not applicable; and −1 = increased. The total score of the above two items was calculated. Total scores ranged from −2 to 2, with higher scores indicating a positive change (decreased alcohol consumption and smoking).

#### COVID-19-related anxiety

COVID-19-related anxiety was measured using the following five items.

(1) I am concerned about getting infected with COVID-19. (2) I am concerned that I might die if I get infected. (3) I am concerned that I will be criticized or harmed by those around me. (3) I am concerned that those who are immunocompromised or my family members will become infected. (4) I am concerned about financial hardships.

Each question was rated on a 5-point Likert scale (1 = very to 5 = not at all), and the total score was calculated by an inverse conversion. Scores ranged from 5 to 25 points, where a higher score indicated a higher level of anxiety. Cronbach's alpha in the present study was 0.80.

#### Social factors

Social factors included social encounters and social support pertaining to the context of COVID-19. COVID-19-related social encounters was examined using the following item: “What kind of changes have happened in your frequency in engaging with your friends or neighbors compared with prior to the COVID-19 pandemic?” The response options were as follows: 1=increased; 2=similar; and 3=decreased. The responses “increased” or “similar” were classified as “not decreased.” The response “decreased” was classified as “decreased.”

Social support was measured as the number of people the participant could ask for help if quarantining due to COVID-19. The number of people was classified as 0, 1–2, or 3 or more.

#### General characteristics

General characteristics including gender, age, education level, monthly household income, employment, living arrangements, and subjective health status were recorded. Age was categorized as younger than 40 years, 40–64 years, and 65 years or older. Education level was categorized into “middle school or less” and “high school or higher.” Monthly household income was categorized as follows: under 1,000,000 KRW; 1,000,000–2,990,000 KRW; 3,000,000–4,990,000 KRW; and 5,000,000 KRW or over. Employment was categorized into “employed” or “unemployed.” Living arrangements were categorized into “living alone” or “living with others.” Subjective health status was categorized as “good” (very good or good), “fair” (fair), or “poor” (poor or very poor).

#### Data analysis

The data were analyzed using a complex sample design using the SPSS/WIN 23.0 program. A descriptive statistical analysis was conducted on the measured variables. The changes in lifestyle and COVID-19-related anxiety according to the general participant characteristics were analyzed using *t*-tests and one-way ANOVA, followed by a *post-hoc* Bonferroni test (Aim 1). A multiple regression analysis was conducted to identify the factors affecting lifestyle changes (Aim 2). Statistical significance was defined as *p* < 0.05.

## Results

### General characteristics and social factors

Among the participants, 49.9% were male, and 50.1% were female. The average age was 48.32 years. Approximately 81.4% of the participants had an educational level of high school or higher. An average monthly household income of 5,000,000 KRW or higher was most common at 37.4%. Regarding living arrangements, 87.9% of participants were living with others. Finally, 53.4% of the participants reported having “good” subjective health status (Table 1).

**Table 1:** General characteristic and social factors of the participants (*N* = 213,848).

**Variables**	**Category**	**Unweighted *n***	**Weighted %**
		**or M** ±**SE**	
Gender	Male	97,524	49.9
	Female	116,324	50.1
Age (years)	19–39	49,274	33.7
	40–64	98,462	47.3
	≥65	66,112	18.9
		48.32 ± 0.054	
Education level	≤Middle school	69,220	18.6
	≥High school	144,628	81.4
Household income (units: KRW 1,000 won/month)	<1,000	33,141	8.8
	1,000–3,000	66,670	25.9
	3,000–5,000	53,259	27.9
	≥5,000	60,778	37.4
Employment	No	81,803	37.1
	Yes	132,045	62.9
Living arrangement	Living alone	33,259	12.1
	Living with others	180,589	87.9
Subjective health status	Good	103,924	53.4
	Fair	82,675	37.7
	Poor	27,249	8.9
Anxiety of COVID-19		19.281 ± 0.013	
Social encounters due to COVID-19	Not decreased	28,463	10.4
	Decreased	185,385	89.6
Number of people to ask for help	0	36,032	15.1
	1–2	93,694	44.5
	≥3	84,122	40.3
Lifestyle change		−0.241 ± 0.004	
Healthy behavioral change index		−0.506 ± 0.003	
Addictive behavioral change index		0.265 ± 0.002	

The average score on COVID-19-related anxiety was 19.28 out of 25. Regarding social support, 44.5% of the participants reported having 1–2 individuals to ask for help during COVID-19 quarantine, 40.3% had 3 or more individuals, and 15.1% had none. In addition, 89.6% of the participants responded that their social encounters had decreased due to COVID-19. The mean scores on the Healthy Behavioral Change Index and Addictive Behavioral Change Index were −0.51 and 0.27, respectively (Table 1).

### Healthy behavioral change index and addictive behavioral change index

Healthy Behavioral Change Index scores, which assessed physical activity, sleep, and unhealthy diet, were higher in male than female, higher in older adults (≥65) than in their younger counterparts, higher in those with an educational level of middle school or than in those with an education level of high school or higher, higher in those with a lower monthly household income (< 1,000,000 KRW) than in those with a higher monthly household income, higher in unemployed individuals than in employed individuals, higher in individuals with a good subjective health status than in those with a poor subjective health status, higher in those living with others than in those living alone, higher in those who did not experience a decrease in social encounters than in those who did, and higher in those who had more than 3 individuals to ask for help than in those who had 1–2 individuals (Table 2).

**Table 2:** Healthy behavioral and addictive behavioral changes.

**Variables**	**Category**	**Healthy behavioral change index**			**Addictive behavioral change index**		
		**M ±SE**	**T or** **modified** **Wald F**	***p***	**M ±SE**	**T or** **modified** **Wald F**	***p***
Gender	Male	−0.233 ± 0.005	29.298	<0.001	0.241 ± 0.004	45.555	<0.001
	Female	−0.384 ± 0.005			0.081 ± 0.003		
Age (years)	19–39^a^	−0.638 ± 0.005	1305.664	<0.001	0.313 ± 0.004	263.881	<0.001
	40–64^b^	−0.499 ± 0.004	a <b <c		0.254 ± 0.003	a>b>c	
	≥65^c^	−0.287 ± 0.004			0.207 ± 0.003		
Education level	≤Middle school	−0.177 ± 0.006	44.444	<0.001	0.132 ± 0.004	−14.880	<0.001
	≥High school	−0.439 ± 0.005			0.189 ± 0.003		
Household income (unit: KRW 1,000 won/month)	<1,000^a^	−0.359 ± 0.007	231.130	<0.001	0.195 ± 0.005	134.027	<0.001
	1,000–3,000^b^	−0.435 ± 0.005	a>b>c>d		0.234 ± 0.003	a<b<c<d	
	3,000–5,000^c^	−0.541 ± 0.006			0.263 ± 0.004		
	≥5,000^d^	−0.564 ± 0.005			0.305 ± 0.003		
Employment	No	−0.292 ± 0.005	5.753	0.003	0.149 ± 0.004	−6.311	<0.001
	Yes	−0.325 ± 0.005			0.172 ± 0.003		
Living arrangement	Living alone	−0.325 ± 0.007	−4.400	<0.001	0.156 ± 0.005	−1.827	0.068
	Living with others	−0.291 ± 0.004			0.166 ± 0.003		
Subjective health status	Good^a^	−0.487 ± 0.004	34.664	<0.001	0.298 ± 0.003	284.615	<0.001
	Fair^b^	−0.535 ± 0.005	b<c, a		0.241 ± 0.003	a>b>c	
	Poor^c^	−0.500 ± 0.008			0.169 ± 0.005		
Social encounters due to COVID-19	Not decreased	−0.152 ± 0.007	42.453	<0.001	0.059 ± 0.005	−41.745	<0.001
	Decreased	−0.465 ± 0.004			0.262 ± 0.003		
Number of people to ask for help	0^a^	−0.491 ± 0.007	10.198	<0.001	0.200 ± 0.004	158.320	<0.001
	1–2^b^	−0.519 ± 0.004	b<c, a		0.262 ± 0.003	a<b<c	
	≥3^c^	−0.497 ± 0.005			0.294 ± 0.003		

*Post hoc, Bonferroni test (a–d = subgroups of each variable).

Addictive Behavioral Change Index scores were higher in male than female, higher in those with a good subjective health status than in those with a poor subjective health status, and higher in those with a greater number of individuals to ask for help than in those with fewer social contacts, indicating that these groups had a lower level of alcohol consumption and smoking than the others. In contrast to the Health Behavioral Change Index scores, decreased alcohol consumption and smoking were observed in younger individuals (aged 19–34 years), those with a higher education level, those with a monthly household income at or > 5,000,000 KRW, employed individuals, and those who experienced a decrease in social encounters (Table 2).

### COVID-19-related anxiety

COVID-19-related anxiety was higher in female than male, higher in older adults (aged ≥65 years) than their younger counterparts, higher in those with a lower education level (middle school or lower) than in those with a higher education level, higher in those with a low monthly household income (< 1,000,000 KRW) than in those with a high monthly household income, higher in employed individuals than in unemployed individuals, higher in those with a bad subjective health status than in those with a good subjective health status, higher in those who living with others than in those living alone, higher in those who experienced a decrease in social encounters than in those who did not, and higher in those with fewer individuals to contact for help than in those with more social contacts (Table 3).

**Table 3:** COVID-19-related anxiety.

**Variables**	**Category**	**COVID-19-related anxiety**		
		**M ±SE**	**T or** **modified** **Wald F**	***p***
Gender	Male	18.745 ± 0.025	−45.622	<0.001
	Female	19.688 ± 0.024		
Age (years)	19–39^a^	18.637 ± 0.022	1372.465	<0.001
	40–64^b^	19.331 ± 0.017	(a<b<c)	
	≥65^c^	20.284 ± 0.023		
Education level	≤Middle school	19.965 ± 0.027	57.081	<0.001
	≥High school	18.468 ± 0.024		
Household income (units: KRW 1,000 won/month)	<1,000^a^	19.965 ± 0.035	323.830	<0.001
	1,000–3,000^b^	19.651 ± 0.023	(a>b>c>d)	
	3,000–5,000^c^	19.268 ± 0.023		
	≥5,000^d^	18.872 ± 0.023		
Employment	No	19.192 ± 0.026	−2.118	0.034
	Yes	19.241 ± 0.024		
Living arrangement	Living alone	19.017 ± 0.033	−12.180	<0.001
	Living with others	19.416 ± 0.021		
Subjective health status	Good^a^	18.965 ± 0.018	819.482	<0.001
	Fair^b^	19.448 ± 0.018	(a<b<c)	
	Poor^*c*^	20.448 ± 0.033		
Social encounters due to COVID-19	Not decreased	18.751 ± 0.037	−24.600	<0.001
	Decreased	19.682 ± 0.017		
Number of people to ask for help	0^a^	19.844 ± 0.030	529.885	<0.001
	1–2^b^	19.479 ± 0.017	(a>b>c)	
	≥3^c^	18.850 ± 0.019		

*Post hoc, Bonferroni test (a–d = subgroups of each variable).

### Factors influencing the healthy behavioral change index and addictive behavioral change index

**Figure 1:**
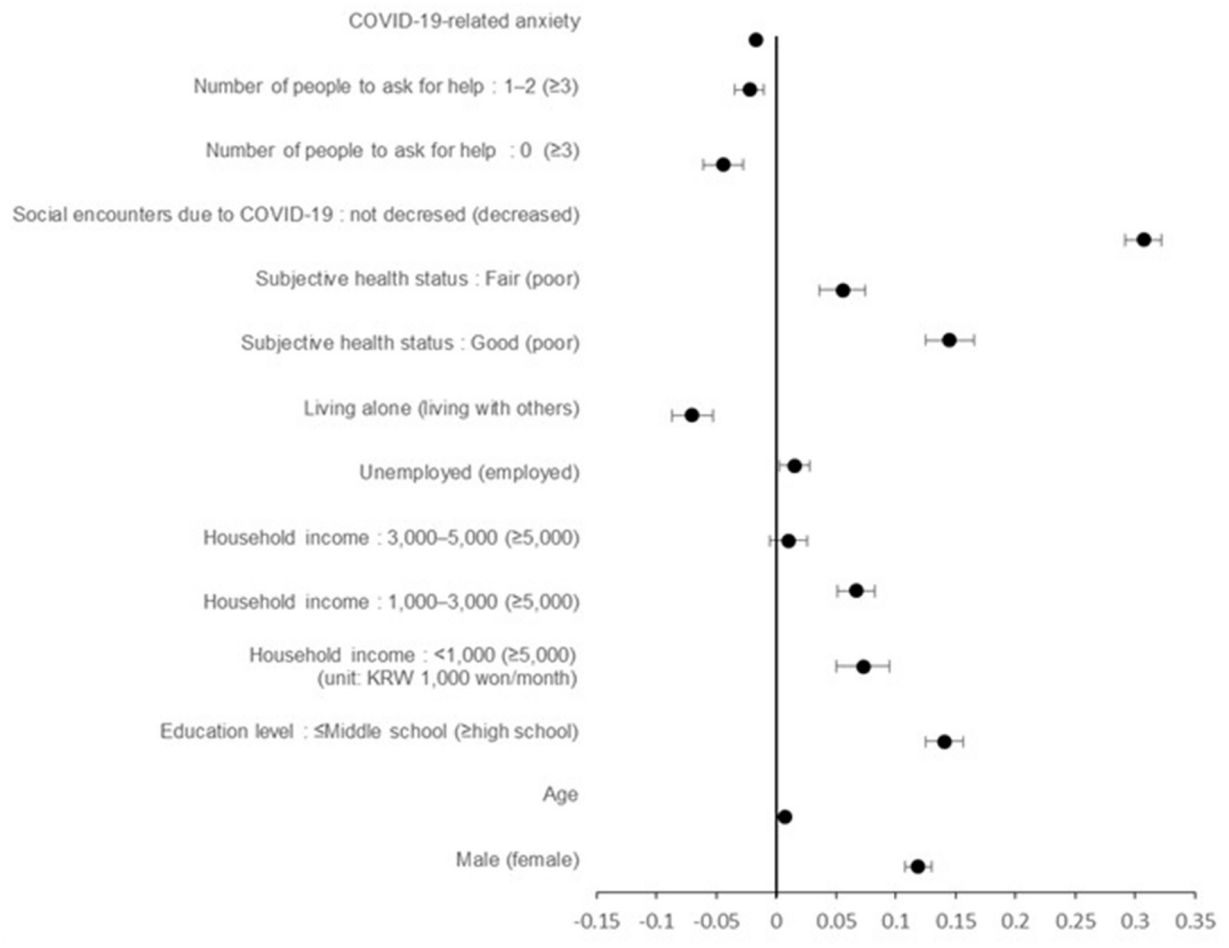
Forest plot of healthy behavioral change index related factors.

Negative changes in health behaviors occurred with higher levels of COVID-19-related anxiety (B = −0.017, 95% CI = −0.018 to −0.015), in individuals living alone (B = −0.070, 95% CI= −0.087 to −0.05), and in those with fewer individuals to ask for help (B = −0.044, 95% CI = −0.061 to −0.027). Namely, their physical activity levels and sleep duration decreased and unhealthy eating increased. On the other hand, health behaviors increased in male (B = 0.119, 95% CI = 0.108 to 0.130), older participants (B = 0.007, 95% CI = 0.006 to 0.007), those with a lower education level (middle school or less) (B = 0.141, 95% CI = 0.125 to 0.156), those with an average monthly household income of < 1,000,000 KRW (B = 0.073, 95% CI = 0.050 to 0.095), unemployed individuals (B = 0.016, 95% CI = 0.003 to 0.02), those with good subjective health status (B = 0.145, 95% CI = 0.125 to 0.165), and those who did not experience a decrease in social encounters (B = 0.307, 95% CI = 0.292 to 0.322) (Table 4; Figure 1).

**Table 4:** Factors influencing the healthy behavioral change index and addictive behavioral change index.

**Variables** **comparison (reference)**	**Healthy behavioral change index**				**Addictive behavioral change index**			
	**B**	**95% CI**		***p***	**B**	**95% CI**		***p***
Gender								
Male (female)	0.119	0.108	0.130	<0.001	0.167	0.160	0.175	<0.001
Age	0.007	0.006	0.007	<0.001	−0.002	−0.002	−0.002	<0.001
Education level								
≤Middle school (≥High school)	0.141	0.125	0.156	<0.001	0.016	0.006	0.026	0.002
Household income (units: KRW 1,000 won/month)								
<1,000 (≥5,000)	0.073	0.050	0.095	<0.001	−0.018	−0.033	−0.002	0.026
1,000–3,000 (≥5,000)	0.067	0.051	0.083	<0.001	−0.032	−0.043	−0.021	<0.001
3,000–5,000 (≥5,000)	0.011	−0.005	0.026	0.169	−0.031	−0.041	−0.021	<0.001
Employment								
Unemployed (Employed)	0.016	0.003	0.028	0.014	−0.010	−0.018	−0.003	0.007
Living arrangement								
Living alone (Living with others)	−0.070	−0.087	−0.053	<0.001	0.002	−0.010	0.014	0.769
Subjective health status								
Good (poor)	0.145	0.125	0.165	<0.001	0.057	0.044	0.070	<0.001
Fair (poor)	0.056	0.036	0.075	<0.001	0.025	0.012	0.037	<0.001
Social encounters due to COVID-19								
Not decreased (decreased)	0.307	0.292	0.322	<0.001	−0.209	−0.218	−0.199	<0.001
Number of people to ask for help								
0 (≥3)	−0.044	−0.061	−0.027	<0.001	−0.055	−0.065	−0.044	<0.001
1–2 (≥3)	−0.022	−0.034	−0.010	<0.001	−0.010	−0.018	−0.002	0.011
COVID-19-related anxiety	−0.017	−0.018	−0.015	<0.001	0.000	−0.001	0.001	0.386

**Figure 2:**
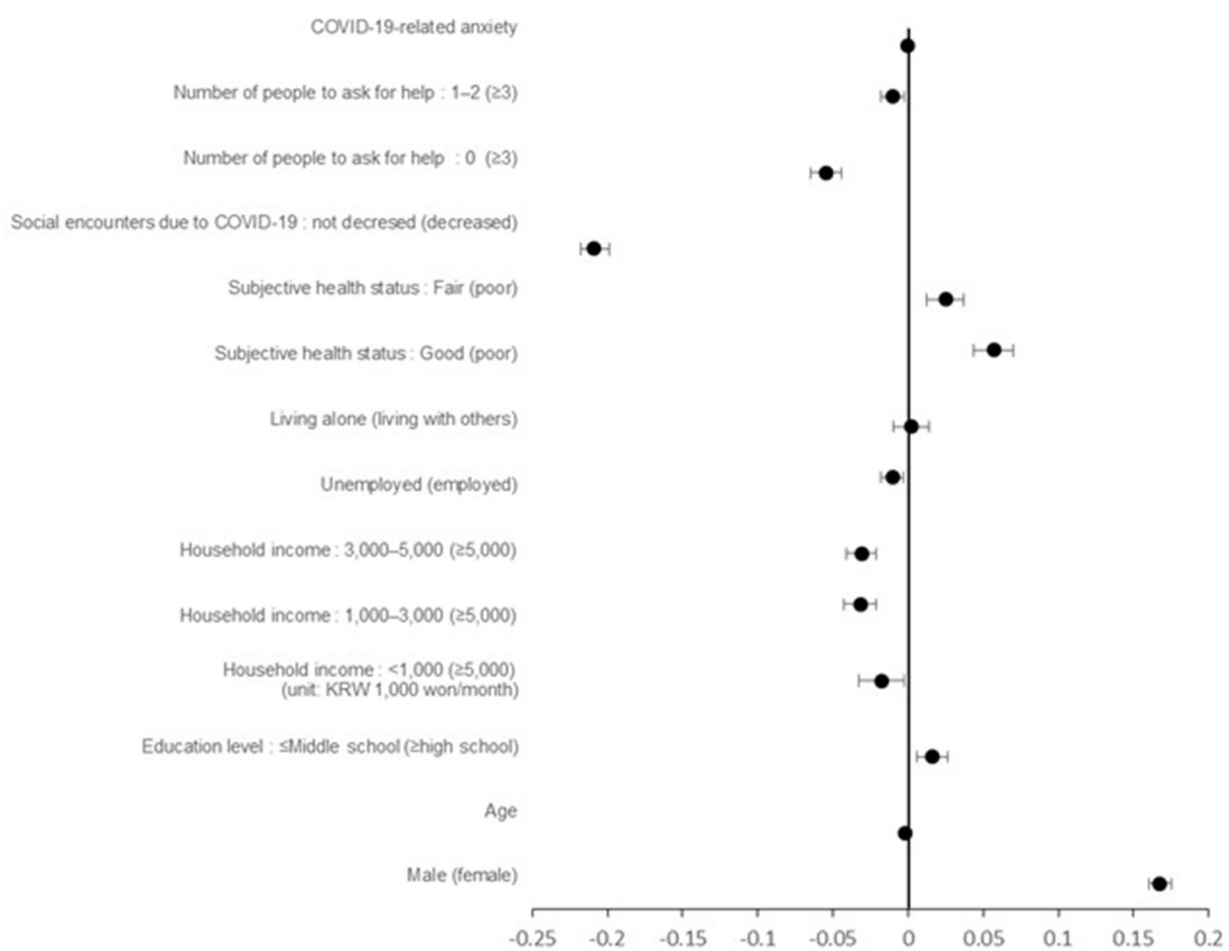
Forest plot of addictive behavioral change index related factors.

In addictive behaviors, alcohol consumption and smoking decreased in male (B = 0.167, 95% CI = 0.160 to 0.175), those with a lower education level (middle school or less) (B = 0.016, 95% CI = 0.006 to 0.026), and those with a good subjective health status (B = 0.057, 95% CI = 0.044 to 0.070). On the other hand, alcohol consumption and smoking increased in older individuals (B = −0.002, 95% CI= −0.002 to −0.002), those with higher average monthly household income (B = −0.031, 95% CI= −0.041 to −0.021), unemployed individuals (B = −0.010=, 95% CI = −0.018 to −0.003), those with fewer individuals to ask for help (B = −0.055, 95% CI = −0.065 to −0.044), and those who did not experience a decrease in social encounters (B = −0.209, 95% CI = −0.218 to −0.199). Unlike the Healthy Behavioral Change Index, addictive behavior was not significantly associated with COVID-19-related anxiety (B = 0.000, 95% CI = −0.001 to 0.001) (Table 4; Figure 2).

## Discussion

This study aimed to examine Koreans' COVID-19-related anxiety and lifestyle changes throughout the COVID-19 pandemic and to identify factors affecting these lifestyle changes.

First, participants demonstrated a high level of COVID-19-related anxiety (19.28 out of 25 points) in this study. No previous studies have used the COVID-19-related-anxiety scale used in this study. Although it is difficult to directly compare with the results of previous studies, this finding is similar to those of other studies conducted in China, the US, and the UK that reported an increase in anxiety due to COVID-19 uncertainties (10, 12–14, 22). From these studies, people showed high levels of anxiety during the COVID-19 pandemic. High levels of anxiety were significantly associated not only with age but also with social factors. Increased levels of COVID-19-related anxiety were observed in older adults, which was similar to the findings of previous studies (15–17). Older adults are more vulnerable to infection and have higher rates of hospitalization and mortality than younger adults due to an increased risk of severe illness following infection (23, 24). These are reasons why older adults may have had increased COVID-19-related anxiety. Furthermore, older adults may experience fear of COVID-19 due to misinformation (23). Thus, it may be necessary to assess their health literacy, which refers to the ability to access and understand accurate information. As a result of this study, increased levels of anxiety were related to lower levels of social support or fewer social encounters. This finding is similar to those of previous studies that have demonstrated decreased anxiety with increased perceived social support (14, 17). It is important to assess anxiety levels and isolation of older adults or individuals with weak social support to detect high-risk groups. However, this study only dealt with COVID-19-related anxiety. Future studies are needed to use a proper diagnostic tool in order to identify specific mental health problems such as anxiety or depression.

Second, this study showed that the Healthy Behavioral Change Index scores negatively changed to −0.51. This result supports the findings of previous studies on negative changes in physical activity (3–6), dietary habits (3, 6) and sleeping patterns (3, 6, 7) during the COVID-19 pandemic.

In this study, high levels of COVID-19-related anxiety were associated with decreased physical activity, such as walking and exercising, increased consumption of unhealthy food, such as instant food or carbonated beverages, and decreased sleep duration. Mental health, such as anxiety and depression, and physical health are interrelated (6). Findings that psychological distress involving anxiety has a negative impact on health behavior support the findings of this study (18, 19). High level of COVID-19-related anxiety can lead to maladaptive coping such as avoidance, rumination, suppression, etc. (25). For this reason, there is a possibility that people with higher level of COVID-19- related anxiety have reduced social contact more excessively than the quarantine standards. More delivered food intake and less outside activities may result in unhealthier behaviors.

Negative lifestyle changes such as increased unhealthy diet and decreased physical exercise can worsen well-being (26). Therefore, these findings indicate a need for interventions to reduce COVID-19-related anxiety.

Our findings showed that in addition to COVID-19-related anxiety, a decrease in social encounters was associated with negative changes in health behavior. In terms of dietary lifestyle, the consumption of delivered or take-out instant foods and unhealthy food increased as the number of outings and social encounters decreased with the onset of the COVID-19 pandemic. A previous study reported that Korea has a social environment in which services such as food delivery applications are well-developed (27), which could have led to an increase in food deliveries following the onset of the pandemic, and that most delivered foods were unhealthy options such as fast food (28). Changes in eating habits due to the COVID-19 pandemic have manifested differently in different nations. An American study, for instance, reported an increase in healthy food consumption during the COVID-19 pandemic (8). This increase in the consumption of healthy food may be attributed to more time being spent at home and cooking (8). Fast food consumption will likely continue to increase among Koreans with the prolonged COVID-19 pandemic. Fast food may have negative impacts on health, such as obesity from high caloric intake (29). Thus, interventions to correct poor dietary habits are urgently needed.

This study found that negative changes also occurred in physical activity and sleeping patterns. This finding was similar to those of previous studies that suggested that physical activity levels have decreased as people stayed at home (4), which in turn has had a negative impact on sleeping patterns (6). During the COVID-19 pandemic, home-based online exercise programs have developed, and this can be helpful to promote healthy lifestyles (30). Based on these results, increasing awareness of the need for regular exercise and actively making online workouts programs that can be done at home available are necessary.

The findings of this study showed that negative changes in physical activity, diet, and sleeping behavior were more likely for individuals who are living alone or do not have others to ask for help. This implies that social factors have an effect on health behavior. This result supports the findings of previous studies that suggest that having more family members (5) and increased social support (19) promote positive changes in health behavior. Thus, developing interventions that help to maintain social networks while complying with social distancing guidelines is important.

However, this study used cross-sectional data which was collected since the onset of the COVID-19 pandemic. It is careful to make a conclusion that these influencing factors will persist once COVID-19-related anxiety abates. Therefore, a longitudinal study is necessary to identify the factors affecting health behavior in the post-COVID-19 era.

Third, a score of 0.27, indicating a slightly positive change, was noted for addictive behavior such as smoking or alcohol consumption. Namely, rates of smoking and drinking decreased in this study. This finding is supported by those of previous studies that indicated a decrease in alcohol consumption (5, 7) and an increase in decisions to quit smoking or reduce smoking frequency (31) during the COVID-19 pandemic. In this study, unlike the factors affecting health behavior, addictive behavior was not associated with COVID-19-related anxiety. Addictive behaviors were negatively related in cases in which social encounters did not decrease.

This finding implies that alcohol consumption or smoking are everyday habits that were not–or were very rarely–affected by COVID-19. Rather, decreased alcohol consumption was likely observed with a reduction in social encounters during the COVID-19 pandemic because of the decrease in social drinking since social activities were restricted by the lockdowns (32). Addictive behaviors such as smoking or alcohol consumption can have a negative impact on the severity of COVID-19 (33). In particular, cigarette smoking is a risk factor for respiratory diseases, is closely associated with an adverse disease prognosis, and is known to impact COVID-19 outcomes (including severe symptoms, negative progression of symptoms and increased mortality) (34). The dangers of cigarette smoking have thus been widely publicized at the national level (35, 36), which may have resulted in a decrease in smoking behavior during the COVID-19 pandemic.

The negative changes in healthy behaviors were related to the more educated, the more employed, and the higher income groups. Yet the same groups showed positive changes in addictive behaviors. These results are different from previous studies that reported the association between high socioeconomic levels and positive health behaviors (37). This inconsistence may be due to the fact that higher socioeconomic levels may increase the chances of engaging in unhealthy behaviors under the specific COVID-19 pandemic.

Healthy behaviors may be related to economic status. Consumption of delivery food requires high costs. The consumption of delivered foods was associated with higher income and the more educated groups in Korea (38). Most delivered foods were unhealthy options such as fast food and high-caloric food (28). We also could expect that higher income and higher level of education groups can address their needs by staying at home as a protective action against COVID-19. The higher rate of working from home is related to higher income and a more stable job (39). Working from home can lead to a reduction in exercise (40).

On the other hand, addictive behaviors were associated with positive changes in the same groups in this study. Social drinking is likely to decrease as social activity decreases (32). In a higher socioeconomic position, they can have a chance to work from home (39), so this may be the result of a decrease in face-to-face behavior and social drinking. However, future research is needed to identify the exact reasons for the unusual results.

Although many countries around the world are preparing for a “post-COVID-19 era,” as the pandemic prevails, COVID-19-related anxiety is still high. This study demonstrates that COVID-19-related anxiety has negatively impacted health behaviors involving physical activity, diet, and sleep. Older adults and those with lower social support were identified as high-risk groups. This study is significant as its findings can guide the direction of health promotion interventions for the upcoming post-COVID-19 era. Nevertheless, this study has some limitations. First, it used cross-sectional data, which makes it impossible to determine an exact causal relationship between anxiety and health behavior. Second, we used COVID-19-related anxiety scale. This is not standardized and has not been validated in previous studies. The future study is suggested to use the scale that verified the reliability and validity. Third, the effect that decreased health behaviors have on health status and the relationship between these two factors could not be identified.

## Conclusion

This study aimed to identify factors affecting lifestyle changes focusing on COVID-19-related anxiety. The findings of this study indicate that anxiety and decreased social encounters due to COVID-19 have resulted in negative changes in health behaviors involving physical activity, diet, and sleep. Based on these results, the following suggestions can be made. First, modification of health behaviors is needed, especially targeting those with negative changes related to COVID-19. Second, given that there are high levels of COVID-19-related anxiety among older adults and those with lower social support, health care providers should make an effort to identify high-risk groups for deteriorating health behaviors early. Furthermore, developing interventions for individuals who are vulnerable to anxiety is necessary. In addition, considering ways to improve health literacy in older adults or maintain and strengthen social networks for online communication is important. The practice of health behaviors must be encouraged, particularly through virtual programs such as the distribution of home-based online workout and education programs on the importance of proper calorie and nutrient intake. Finally, long-term studies on the effect of changes in health behavior throughout the whole COVID-19 era are needed along with lifestyle changes due to COVID-19.

## Data availability statement

The original contributions presented in the study are included in the article/supplementary material, further inquiries can be directed to the corresponding author.

## Ethics statement

The studies involving human participants were reviewed and approved by Institutional Review Board of the Gachon University (IRB No. 1044396-202111-HR-227-01). The patients/participants provided their written informed consent to participate in the 2020 Korean Community Health Survey.

## Author contributions

SYH, HYJ, and YK were responsible for the study conception and design and responsible for the drafting of the manuscript. SYH and YK performed the data analysis and supervised the study. HYJ provided statistical expertise. SYH provided administrative, technical, material support, and wrote the first draft. All authors read and approved the final manuscript.

## Conflict of interest

The authors declare that the research was conducted in the absence of any commercial or financial relationships that could be construed as a potential conflict of interest.

## Publisher's note

All claims expressed in this article are solely those of the authors and do not necessarily represent those of their affiliated organizations, or those of the publisher, the editors and the reviewers. Any product that may be evaluated in this article, or claim that may be made by its manufacturer, is not guaranteed or endorsed by the publisher.
